# Integrated Wastewater Remediation and Energy Production: Microfluidic Photocatalytic Fuel Cells Enabled by Dye Pollutants

**DOI:** 10.3390/mi16030312

**Published:** 2025-03-07

**Authors:** Youquan Zhou, Fangzhou Luo, Zhichao Wang, Jiayi Zhu, Hao Yang

**Affiliations:** 1 National Engineering Research Center of Fiber Optic Sensing Technology and Networks, Wuhan University of Technology, Wuhan 430070, China; zyquan0411@whut.edu.cn (Y.Z.); 294552@whut.edu.cn (F.L.); 122600454@163.com (Z.W.); zhujy@whut.edu.cn (J.Z.); 2 Hubei Optical Fundamental Research Center, Wuhan 430074, China; 3 Wuhan Fibers Technology Co., LTD, Wuhan 430223, China

**Keywords:** photocatalytic fuel cells, RhB, antibiotic degradation

## Abstract

Directly degrading the dyes in the wastewater is a missed opportunity. Herein, we propose a solution employing a microfluidic chip to construct a photocatalytic fuel cell (PFC) system, which can efficiently degrade tetracycline while generating electricity simultaneously under visible-light irradiation. This approach utilizes the photogenerated electrons from the dye Rhodamine B (RhB), which are effectively transferred through a gold layer to activate persulfate in water, leading to enhanced tetracycline degradation. Experimental results reveal that within one hour of reaction duration, the degradation efficiency of tetracycline within the PFC system was doubled. At a persulfate (PS) concentration of 2 mM, the system’s open-circuit voltage and short-circuit photocurrent density reached 0.26 V and 0.00239 mA·cm^−2^ respectively, both exceeding the values detected at 0.5 mM PS. Additionally, the system’s power density was triple that at 0.5 mM PS. Notably, when the PS concentration in the system was elevated from 0.5 mM to 2 mM, the degradation efficiency of tetracycline witnessed a significant boost from 35.16% to 60.78%. This approach proffers a novel tactic for harnessing dye waste via microfluidic devices. The PFC system accomplishes not only the degradation of dyes and antibiotics but also the generation of electrical energy, substantially enhancing the energy utilization efficiency.

## 1. Introduction

The urgent challenges of wastewater treatment and energy shortage [1,2] necessitate the exploration of new technologies for effective solutions. Currently, photocatalytic technology [3,4] has emerged as a promising means to substantially enhance the efficiency of environmental pollution remediation and clean energy production. Among them, the photocatalytic fuel cell (PFC) [5,6,7] represents an advanced technology that employs photocatalysis to retrieve energy from wastewater. PFCs utilize the photogenerated electron–hole pairs within photocatalytic materials to prompt electrochemical redox reactions [8,9], facilitating the concurrent treatment of pollutants and electricity generation. This integrated system not only tackles water pollution but also facilitates renewable energy generation, rendering it an appealing solution to the dual challenges of wastewater management and energy shortage.

Currently, optofluidic technology [10,11] has emerged as a promising approach for fabricating photocatalytic fuel cell (PFC) devices and augmenting photocatalytic activity. This is due to its larger reaction surface area and shorter light propagation distance, facilitating photon transmission [12,13]. Researchers are increasingly integrating photocatalytic fuel cells (PFCs) with microfluidic devices to boost device performance. For instance, Li et al. [10] investigated glucose degradation as a model pollutant in microfluidic photocatalytic fuel cells, utilizing TiO_2_/FTO as the anode and a Pt/CP air-breathing cathode. They reported a degradation efficiency of 83.9% and a maximum power density of 0.58 mW·cm^−2^. Nevertheless, compared with traditional approaches entailing the deposition of composite materials on battery electrodes to utilize the photogenerated electron–hole pairs [14,15], a novel microfluidic PFC system with direct in-situ reaction in a reaction chamber provides a more direct and convenient method for simultaneous degradation of pollutants and power generation. Within this microfluidic PFC system, the photocatalytic dye waste is directly incorporated into the reaction solution, obviating the necessity for complex material fabrication and electrode modifications [16,17]. The photogenerated electrons and holes can partake in the redox reactions within the microfluidic channels, expediting the degradation of target pollutants like antibiotics and concurrently generating an electrical current available for harvesting. The direct integration of the photocatalytic process and electrochemical reactions within the microfluidic platform offers multiple advantages. First, the photocatalytic reaction and electrochemistry are combined through a microfluidic device. This not only achieves a synergistic effect between photocatalysis and electrochemistry but also optimizes the overall operational complexity to a certain extent. Moreover, the continuous flow and regulated residence time within the microfluidic channels can augment mass transfer and reaction kinetics, culminating in enhanced pollutant removal efficiency and power generation performance.

In a reaction system devised for concurrent pollutant degradation and electricity generation via direct in-situ reactions, activated persulfate (PS) oxidation predicated on the principle of sulfate radical (SO_4_^−^·) oxidation has been extensively investigated and implemented in the degradation of organic pollutants, owing to its merits of being economical, highly efficient, environmentally friendly, safe, and stable [18,19,20]. Furthermore, according to previous studies, PS solutions can be photochemically activated by dye solutions, leading to the generation of highly reactive sulfate radicals [21]. These sulfate radicals have been proven effective in efficiently degrading antibiotics in water [22,23,24]. Through integrating the photogeneration of sulfate radicals from persulfate within the microfluidic PFC system, the degradation of target pollutants like antibiotics can be markedly enhanced [25,26]. The photocatalytic dye waste functions not only as the electron donor for electricity generation but also as the photosensitizer for activating the persulfate, engendering a synergistic effect for water decontamination. Compared with conventional photocatalytic systems, where radical generation and pollutant degradation occur stepwise, this approach may address the issue of its low pollutant removal efficiency.

In this study, we devised and fabricated a microfluidic photocatalytic fuel cell (PFC) system that combines the advantages of photocatalytic technology and microfluidic technology. Rhodamine B (RhB), as a typical pollutant, not only acted as an electron donor for power generation but was also the photocatalyst for the PFC system, enabling the utilization of waste. Subsequently, experiments were conducted in the developed PFC system to achieve the simultaneous degradation of the antibiotic tetracycline and power generation. A comprehensive exploration was undertaken to evaluate the impacts of diverse system parameters, namely, flow rate, light intensity, persulfate concentration, and sodium sulfate concentration, on pollutant degradation and power generation performance. Eventually, we examined the continuous power generation potential of the system, illustrating that the microfluidic photocatalytic fuel cell demonstrates relatively stable power generation performance.

## 2. Device Design and Experiment

### 2.1. Device Design and Fabrication

**Figure 1:**
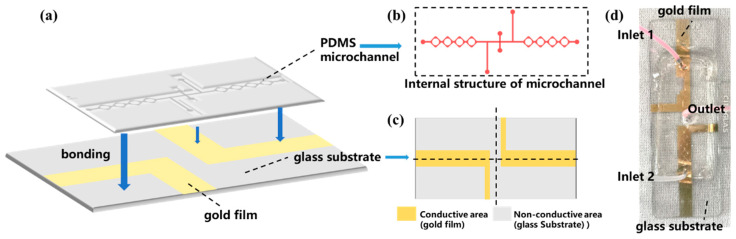
(**a**) The schematic diagram of the PFC, (**b**) the internal structure of the microchannel, (**c**) the design of the glass substrate with gold-plated electrodes, and (**d**) the prepared PFC device.

The schematic diagram of the microfluidic photocatalytic fuel cell is shown in Figure 1a, and its fabrication process is divided into the following steps. First, the internal structure of the microfluidic chip was designed by drawing in Tanner L-Edit software (v 15.0), as shown in Figure 1b. The inlet and outlet on the top indium tin oxide (ITO) glass slide are fabricated to allow the liquid samples to enter the reaction chamber. The rhombic mixing structure was chosen to accelerate the speed of mixing. At the same time, the liquid flow would be accelerated in the connection of each rhombus-shaped mixing unit, and the long flow channel could further promote the mixing of the solutions and the increase in the contact time between the solutions and the electrodes, which could promote the reaction [27].

This microfluidic chip equipped with electrodes has two parts, a microfluidic channel on the top and electrodes on the bottom. The electrodes were fabricated by depositing 5 nm Cr and 30 nm Au onto a glass substrate. The above-designed structural model was fabricated by the standard photolithography. The details are as follows: the wafer is spin-coated with a 50 μm thick photoresist layer. Subsequently, it is exposed to UV lithography, facilitating the reaction and solidification of the exposed photoresist. Eventually, the wafer is cleaned with a developer solution to remove the unexposed photoresist, generating a wafer with a fully designed layout.

PDMS elastomer (about 15 g) and PDMS curing agent (about 1.5 g) were mixed in a ratio of 10:1, stirred for 15 min, and then evacuated in a vacuum oven until the mixture was free of bubbles [28]. Afterward, the mixture was poured onto the photolithographed wafer substrate and heated at about 85 °C for 60 min. After heating, the PDMS material was cured, and the cured PDMS was carefully cut off with a scalpel, and holes were punched at the outlet and inlet with a flat-bladed needle.

Subsequently, the process of electrode preparation was initiated. The initial step involved designing the area and shape of the gold-plated electrodes, guided by the principle that the gold-plated film should be strategically positioned to overlay the microfluidic channel. However, it was crucial to maintain a specific distance (0.5 cm) between the positive and negative electrodes, as illustrated in Figure 1c. The desired electrode shapes were delineated on the glass substrate using adhesive tape, ensuring that the regions requiring gold plating were not covered by the tape. Subsequently, the glass substrate was placed into the magnetron sputtering apparatus, and the necessary parameters were established to achieve the gold-plated glass substrate. The final step involved removing the tape from the substrate, resulting in a glass substrate adorned with a gold-plated film. To facilitate a robust adhesion between the PDMS and the glass substrate, plasma surface activation cleaning was employed to alter the surface characteristics of both materials [29,30]. Ultimately, the microfluidic chip’s inlet and outlet were interfaced with a syringe, and the interfaces between the PDMS and the glass substrate, as well as the syringe and the PDMS, were meticulously sealed employing a UV-curable adhesive, which was subsequently solidified under UV irradiation to ensure leak-proof connections. Finally, the microfluidic photocatalytic fuel cell is obtained, which is shown in Figure 1d.

### 2.2. Materials and Instruments

Rhodamine B (RhB, C_28_H_31_ClN_2_O_3_, reagent grade) was purchased from Shanghai Aladdin Biochemical Technology Co., Ltd. (Shanghai, China). Sulfuric acid (H_2_SO_4_, 98%), potassium persulfate (K_2_S_2_O_8_, AR), and sodium sulfate (Na_2_SO_4_, AR) were purchased from Sinopharm Chemical Reagent Co. (Shanghai, China). Tetracycline (TC, C_22_H_24_N_2_O_8_, CP) was purchased from Shanghai Macklin Biochemical Co., Ltd. (Shanghai, China). PDMS ((C_2_H_6_OSi) _n_, DC184) and curing agent (C_3_H_8_O_2_Si_2_, S184) were purchased from Dow Corning Co. (Midland, MI, USA). All reagents were of analytical grade without special instructions. The deionized water was used in the experiment.

Laser direct writing lithography (uPG501, Heidelberger Druckmaschinen AG), magnetron sputterers, and plasma cleaner (CY-P5L-BT, CY Scientific Instrument Co., Ltd., Zhengzhou, China) were used to fabricate microfluidic photocatalytic fuel cells. A xenon lamp (PLS-SXE300D, Beijing Perfect Light Technology Co., Ltd., Beijing, China) was chosen as the light source for the reaction. An electrochemical workstation (CHI660E, Shanghai CH Instruments Co., Ltd., Shanghai, China) was used to test the electrical properties of the devices. The syringe pump (LSP02-1B, Longer Precision Pump Co., Ltd., Baoding, China) was employed to inject the reagents into the reacting chamber. The UV-Vis spectrophotometer (AvaSpec-ULS2048L, Beijing Avantes Technology Co., Ltd., Beijing, China) was used to measure the absorbance of the RhB and antibiotic samples.

### 2.3. On-Chip Testing

#### 2.3.1. Tetracycline Degradation Performance Evaluation

For the catalytic validation experiments, the reaction solution consisted of 10 mg/L TC, 10 mg/L RhB solution, 1 M sodium sulfate solution (Na_2_SO_4_), and 2 mM potassium persulfate solution (K_2_S_2_O_8_), which was added to a plastic petri dish, and the absorbance at 355 nm of the mixture was measured at A_0_ prior to exposure to light. A xenon lamp was then turned on (fitted with a λ < 400 nm filter). Subsequently, 1 mL samples were extracted every 10 min for absorbance measurement. According to the Lambert–Bill law [31], the degradation efficiency ***η*** of tetracycline is calculated using Equation (1) [32]:

(1) $$\eta =\frac{C_{0}-C_{t}}{C_{0}}\times 100\%=\frac{A_{0}-A_{t}}{A_{0}}\times 100\%$$

where $C_{0}$ and $C_{t}$ represent the initial concentration and the concentration after degradation, respectively. $A_{0}$ is the absorbance at 355 nm of the original tetracycline solution before the degradation, and $A_{t}$ is the absorbance at 355 nm of the solution after the degradation.

For the on-chip test, 1 mL of the 10 mg/L tetracycline solution, 1 mL of the 10 mg/L RhB solution, and 1 mL of the 1 M Na_2_SO_4_ solution were added to inlet 1 to serve as the anodic reaction solution. Meanwhile, 1.5 mL of the 1 M Na_2_SO_4_ solution and 1.5 mL of the 2 mM K_2_S_2_O_8_ solution were added to inlet 2 as the cathodic reaction solution [21]. The syringe was secured in a peristaltic pump, and the initial absorbance (*C*_0_) at 355 nm, corresponding to the maximum absorption peak of tetracycline, was measured after mixing the cathodic and anodic reaction solutions prior to their introduction into the microfluidic chip. Subsequently, the positive and negative electrodes of the battery were wired together, and the syringe was connected to the inlet of the microfluidic chip through a hose. Both the xenon lamp and the peristaltic pump were activated simultaneously. The postreaction solution was then collected at the outlet of the chip, and the absorbance (*C_t_*) at 355 nm was measured. Finally, the tetracycline degradation efficiency was calculated. Under the same conditions, all experiments were repeated three times.

#### 2.3.2. Testing the Power Generation Performance of the System

**Figure 2:**
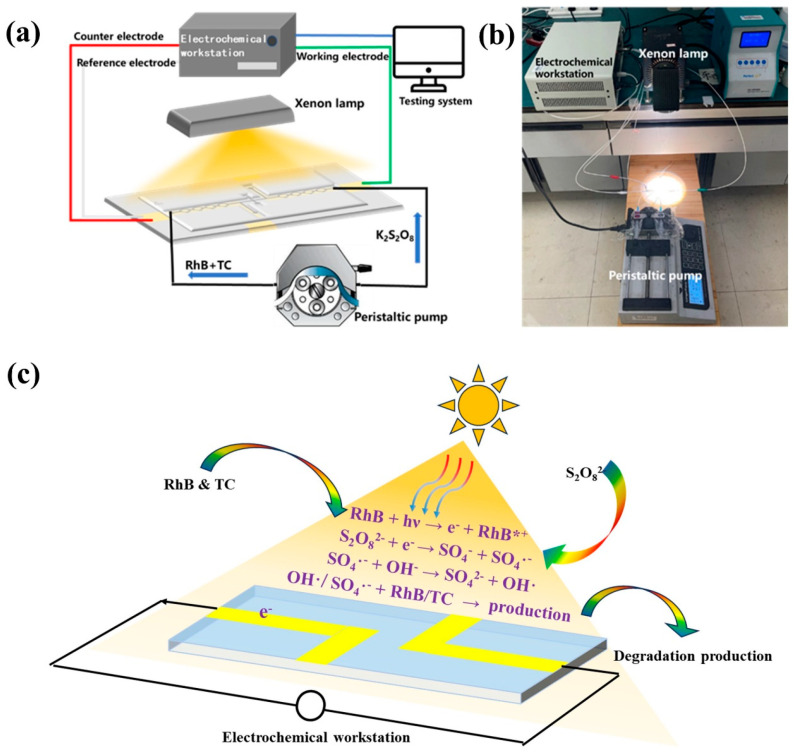
(**a**) Schematic diagram of the test system, (**b**) physical diagram of the test system, and (**c**) the reaction mechanisms of PFC systems.

The working electrode of the electrochemical workstation was connected to the negative pole of the battery, while the reference and counter electrodes were connected to the positive pole. The xenon lamp (equipped with a λ < 400 nm filter) and the peristaltic pump were simultaneously activated, ensuring a consistent inlet rate at both poles to maintain laminar flow. The open-circuit voltage of the system was measured using the electrochemical workstation, and the power generation performance of the system was assessed once the open-circuit voltage stabilized. Figure 2a,b presents the schematic and physical diagrams of the test setup, illustrating that the system primarily comprises two interconnected pathways, which are the test circuit and the liquid path. The test circuit is established by the electrochemical workstation to form a three-electrode system, with battery performance data acquisition facilitated through a computer interface for data reading and storage. In Figure 2a, the three lines in red, green, and white represent the wires connecting the electrodes to the electrochemical workstation. The blue line represents the data cable connecting to the computer, and the black line represents the injection route of the solution. The liquid pathway is configured using a syringe pump, syringe, hose, microfluidic reactor, and a reaction liquid collection device.

The constant potential mode of the test system was employed to obtain the system’s polarization curve and power density curve. In this study, the polarization curves were tested using a constant voltage discharge mode, where the discharge current density of the battery at various voltages was recorded in real-time. For each voltage value, the system reached a stable state within two minutes, so the system was continuously discharged for 120 s, and the current value at the end of this period was taken as the corresponding current value for that voltage. The output power density of the battery can be obtained directly by polarization curve calculation (Equation (2)).

(2) P=UI

where the U is the battery operating voltage (V) and I is the battery current density (A·cm^−2^).

## 3. Experimental Results and Discussion

### 3.1. Validation of the Catalytic Performance of the System

**Figure 3:**
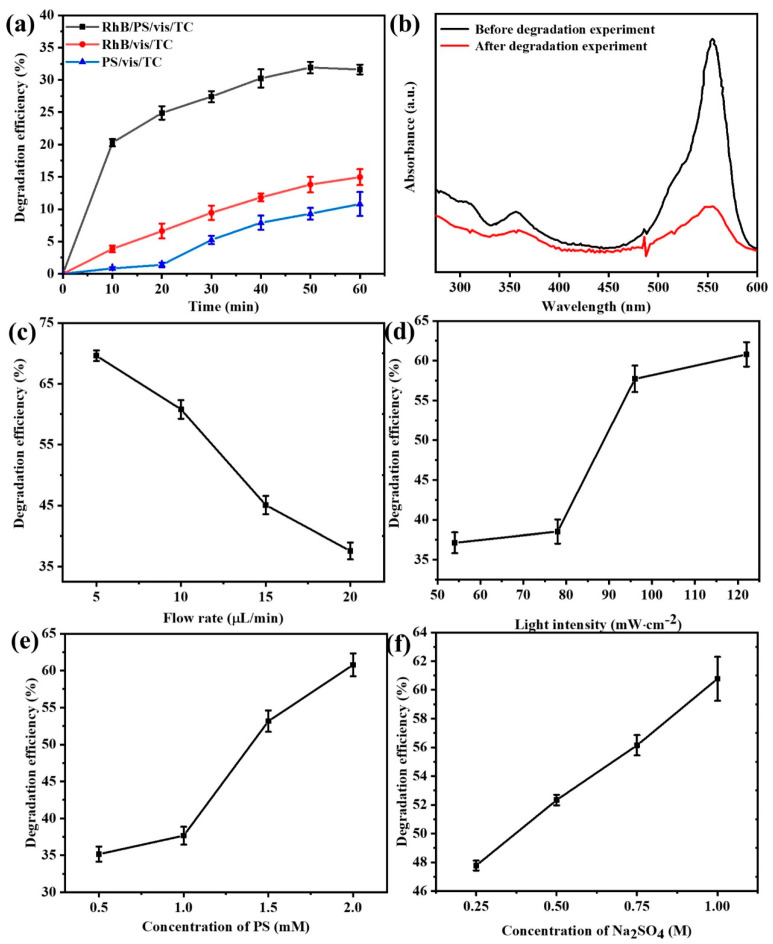
(**a**) The degradation of TC by the photoexcited RhB/PS system, (**b**) the absorption spectra of the solution before and after the reaction, and degradation efficiency of tetracycline at different (**c**) flow rates, (**d**) light intensities, (**e**) PS concentrations, and (**f**) sodium sulfate concentrations in a microfluidic chip.

To explore whether RhB could function as a PS activator for the treatment of antibiotic wastewater, an investigation was conducted on the impacts of photoexcited RhB on the degradation of 10 mg/L TC by PS. First, we conduct preliminary experiments outside the microfluidic chip to determine whether RhB can function as a PS activator. As depicted in Figure 3a, a comparison was made between the degradation of tetracycline by the rhodamine B/K_2_S_2_O_8_/visible light (RhB/PS/vis/TC) system, the rhodamine B/visible light system (RhB/vis/TC), and the K_2_S_2_O_8_/visible light system (PS/vis/TC). Apparently, the RhB/PS/vis/TC system, represented by the black line, exhibited the highest degradation efficiency. Within the initial 5 min reaction period, the degradation efficiency of tetracycline surged to approximately 20%, whereas those of the PS/vis/TC and RhB/vis/TC systems were both below 5%. Eventually, the degradation efficiency of tetracycline by different systems after 60 min of reaction was in the following order: RhB/PS/vis/TC > PS/vis/TC > RhB/vis/TC. More precisely, the degradation efficiency of tetracycline was merely 10.8% in the RhB/vis/TC system and 14.95% in the PS/vis/TC system, whereas it attained 31.61% in the RhB/PS/vis/TC system. These results suggest that neither RhB nor K_2_S_2_O_8_ independently could effectively degrade tetracycline under visible light irradiation, yet their synergistic interaction in the RhB/PS/vis/TC system brought about a substantially enhanced pollutant removal performance.

Figure 3b presents the absorption spectra of the solutions prior to and subsequent to the reaction; the substances in the solution are mainly RhB (Rhodamine B) and TC (Tetracycline). Notably, the maximum absorption peak of the tetracycline solution is located at 355 nm [33], while that of the RhB solution is at 554 nm [34]. From the spectrum, it is evident that RhB also demonstrates certain self-degradation characteristics. In line with the prior report, it was ascertained that RhB could activate PS under visible light irradiation and undergo autocatalytic degradation [35,36], as evidenced by monitoring the alteration of persulfate concentration during the reaction process. Its main degradation pathway is shown as follows [21]:

RhB + hν → e^−^ + RhB^*+^ (3)

S_2_O_8_^2−^ + e^−^ → SO_4_^−^· + SO_4_^2−^ (4)

SO_4_^−^· + OH^−^ → ·OH + SO_4_^2−^ (5)

SO_4_^−^·/·OH + RhB/TC → degradation product (6)

The reaction mechanism and charge transfer pathway of the system were illustrated in Figure 2c. Initially, RhB absorbs light to generate photoexcited electrons and is oxidized to RhB^*+^ (Equation (3)). Subsequently, PS is reduced by the photoexcited electrons, yielding SO_4_^−^· and ·OH (Equations (4) and (5)). Eventually, the resultant free radicals act on the RhB and the tetracycline, inducing their degradation (Equation (6)) [37,38]. During this process, an electric potential difference will arise between the anode and cathode of the system. This potential difference is closely correlated with factors like the system’s flow rate and light intensity. Figure 3c presents the degradation efficiency of tetracycline within the system under varying peristaltic pump feed flow rates, with the light intensity maintained at 122 mW·cm^−2^, the PS concentration held at 2 mM, and the sodium sulfate concentration fixed at 1 M. The degradation efficiency of tetracycline declines progressively from 69.61% to 37.55% as the flow rate is incrementally augmented from 5 μL/min to 20 μL/min. The observed phenomenon can potentially be ascribed to the following: within a given reaction chamber, an elevation in the solution flow rate curtails the time during which the solution engages with the electrode. Consequently, the exposure time to visible light is likewise abbreviated, engendering a fewer number of effective electrons. This, in turn, precipitates a commensurate reduction in the quantity of electrons arriving at the cathode from the external circuit for the reduction reaction with persulfate (Equation (4)). Subsequently, the diminution in activated persulfate attenuates the degradation reaction of tetracycline (Equation (4)), culminating in a diminished degradation efficiency of tetracycline.

Figure 3d presents the tetracycline degradation efficiency within the system under varying light intensities while keeping the flow rate at 10 μL/min, the PS concentration at 2 mM, and the sodium sulfate concentration at 1 M. The tetracycline degradation efficiency augmented from 37.13% to 60.78% as the light intensity was elevated from 54 mW·cm^−2^ to 122 mW·cm^−2^. This can potentially be ascribed to the following: when the light intensity declines, the effective quantity of photons reaching the solution per unit time concomitantly diminishes. Subsequently, the reaction involving RhB (Equation (3)) is enfeebled, giving rise to the generation of fewer electrons. Consequently, PS engenders fewer sulfate radicals via its reduction reaction with electrons, thereby diminishing the efficiency of tetracycline degradation.

Figure 3e illustrates the tetracycline degradation efficiency within the system under diverse concentrations of PS while maintaining the flow rate at 10 μL/min, the light intensity at 122 mW·cm^−2^, and the sodium sulfate concentration at 1 M. The degradation efficiency of tetracycline progressively augmented from 35.16% to 60.78% as the concentration of potassium persulfate was incrementally elevated from 0.5 mM to 2 mM. This can potentially be ascribed to the following: as the concentration of potassium persulfate rises, the reduction reaction between persulfate and electrons is intensified, giving rise to a more copious production of sulfate radicals (SO_4_^−^·), as depicted in Equation (4). Moreover, this augmented persulfate activation expedites a series of reactions implicating sulfate radicals (Equations (5) and (6)), culminating in enhanced degradation efficiency of tetracycline.

Figure 3f displays the tetracycline degradation efficiency within the system under different sodium sulfate concentrations while setting the flow rate at 10 μL/min, the light intensity at 122 mW·cm^−2^, and the PS concentration at 2 mM. As the concentration of sodium sulfate increases from 0.25 M to 1 M, the degradation efficiency of tetracycline gradually improves, rising from 47.77% to 60.78%. Concomitantly, as the concentration of sodium sulfate ascended, the conductivity of the solution inside the chip was fortified, engendering an elevated ion migration rate. This, in turn, directly expedited the advancement of diverse reactions, culminating in a higher tetracycline degradation efficiency.

### 3.2. Effect of Sampling Flow Rate on the Degradation Performance of Tetracycline and Power Generation Performance of the System

**Figure 4:**
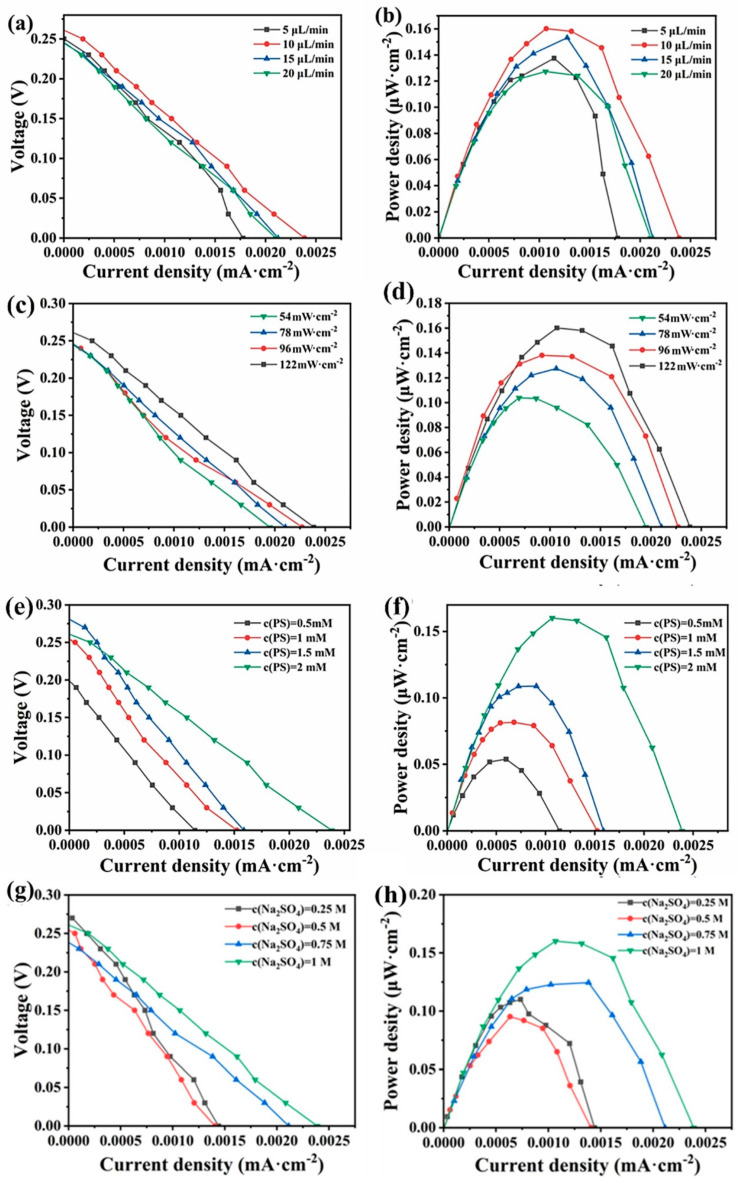
The polarization curve of the system under different (**a**) flow rate, (**c**) light intensities, (**e**) PS concentrations, and (**g**) sodium sulfate concentrations, and the power density curve of the system under different (**b**) flow rates, (**d**) light intensities, (**f**) PS concentrations, and (**h**) sodium sulfate concentrations.

The polarization curves and power density curves of the system at different flow rates were investigated in Figure 4a. When the flow rate was gradually increased from 5 μL/min to 20 μL/min, the open-circuit voltage of the system was basically unchanged, and the open-circuit voltage was relatively maximal at a flow rate of 10 μL/min, which was about 0.26 V. When the flow rate was gradually increased from 5 μL/min to 20 μL/min, the short-circuit current density first increased from the minimum of 0.00178 mA·cm^−2^ to a maximum of 0.00239 mA·cm^−2^ and then gradually decreased to 0.0021 mA·cm^−2^. The power production performance of the photocatalytic fuel cell first increased and then decreased with the increase in the flow rate. The reason may be that at low flow rates, the supply of reactants to the system is insufficient, resulting in a low overall reaction rate. Increasing the flow rate enhances the transport within the cell, resulting in a higher number of electrons passing through the external circuit and improved performance. However, as the flow rate is further increased, the residence time of the solution in the reaction chamber and the interaction with the electrodes decrease. Additionally, excessively high flow rates can promote stronger direct mixing between the cathode and anode electrolytes, which in turn reduces the cell performance. From the polarization curves, the relationship between power density, current, and flow rate can be derived, as illustrated in Figure 4b. It is evident that, similar to the polarization curve, the maximum power density of the cell increases with the flow rate, first from 0.1375 μW·cm^−2^ to a maximum value of 0.16 μW·m^−2^, and then decreases to 0.127 μW·cm^−2^.

Combined with the degradation efficiency of tetracycline and the electrical production performance of the system, 10 μL/min was selected as the optimal injection flow rate of the system.

### 3.3. Effect of Light Intensity on the Degradation Performance of Tetracycline and the Power Generation Performance of the System

The polarization curves and power density curves of the system at different light intensities are investigated in Figure 4c. As shown in Figure 4c, the open circuit voltage of the system increases from 0.24 V to 0.26 V, and the short circuit current density increases from 0.00195 mA·cm^−2^ to 0.00239 mA·cm^−2^ when the light intensity is increased from 54 mW·cm^−2^ to 122 mW·cm^−2^. From the polarization curves, the relationship between power density, current, and flow rate can be derived, as shown in Figure 4d. It is evident that the maximum power density of the cell increases with rising light intensity, reaching 0.103 μW·cm^−2^ at a light intensity of 54 mW·cm^−2^ and 0.16 μW·cm^−2^ at 122 mW·cm^−2^. When the light intensity decreases, the number of photons arriving at the solution per unit of time decreases, and therefore the number of electrons produced per unit of time decreases, resulting in the external circuit current decreasing, and therefore the output power density also decreases. Combined with the degradation efficiency of tetracycline and the system’s power production performance, 122 mW·cm^−2^ was chosen as the optimal light intensity of the system.

### 3.4. Effect of Potassium Persulfate Concentration on Tetracycline Degradation Performance and Power Generation Performance of the System

The polarization curves and power density curves of the system at different potassium persulfate concentrations are investigated. As shown in Figure 4e, when the potassium persulfate concentration was gradually increased from 0.5 mM to 2 mM, the open circuit voltage of the system increased from 0.2 V to 0.26 V and the short-circuit current density increased from a minimum of 0.00114 mA·cm^−2^ to 0.00239 mA·cm^−2^.

From the polarization curves, the curves of the variation of the power density with the current and flow rate were obtained, which are shown in Figure 4f. It is evident that the maximum power density of the cell increases with rising PS concentration. At a PS concentration of 0.5 mM, the maximum power density is 0.0538 μW·cm^−2^, while at a concentration of 2 mM, it reaches 0.16 μW·cm^−2^, representing an enhancement of approximately threefold. This may be attributed to the fact that the elevation of PS concentration augments the electrical conductivity of the solution. Meanwhile, it intensifies its reaction with electrons (Equation (4)), boosts the redox rate, and facilitates the transfer of photogenerated electrons to the cathode via the external circuit, consequently leading to an increase in the current and output power of the system.

Combining the degradation efficiency of tetracycline and the system’s power production performance, 2 mM was chosen as the optimal PS concentration for the system.

### 3.5. Effect of Sodium Sulfate Concentration on Tetracycline Degradation Properties and Power Generation Properties of the System

The polarization curves and power density curves of the system at different sodium sulfate concentrations are investigated. As shown in Figure 4g, when the sodium sulfate concentration is gradually increased from 0.25 M to 1 M, there is no significant change in the open circuit voltage of the system, and the short-circuit current density was increased from a minimum of 0.0014 mA·cm^−2^ to 0.00238 mA·cm^−2^. The curves of variation of the power density with the current and flow rate can be obtained from the polarization curves, as shown in Figure 4h. The power density of the system reached its maximum when the concentration of sodium sulfate was 1 M. In this experiment, the primary role of sodium sulfate is to enhance the solution’s conductivity. Increasing its concentration improves the conductivity within the chip, thereby increasing the collision frequency among the substances in the solution, which promotes the reactions. Additionally, this enhancement boosts the rate of electron transfer in the external circuit, as evidenced by the increase in current and output power.

Considering both the tetracycline degradation efficiency and the system’s power generation performance, a sodium sulfate concentration of 1 M was determined to be optimal for the system.

### 3.6. Continuous Power Generation Performance of the System

**Figure 5:**
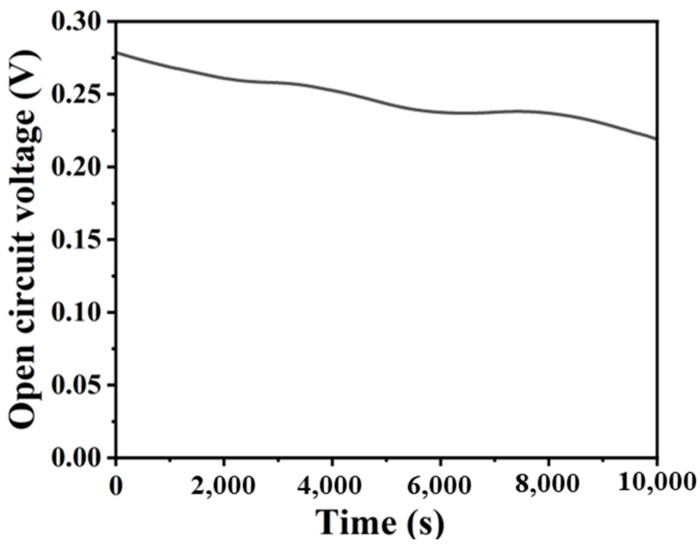
Open-circuit voltage–time plot.

Finally, the continuous discharge performance of the system was tested. As depicted in Figure 5, which illustrates the open circuit voltage variation of the system over 10,000 s (approximately 3 h), the cell voltage remained comparatively stable, with a marginal decline in the voltage value from the initial 0.26 V to 0.236 V. The aforementioned results indicate that the microfluidic photocatalytic fuel cell exhibits relatively stable power generation performance.

## 4. Conclusions

In this study, a microfluidic photocatalytic fuel cell predicated on the advanced oxidation of activated persulfate was devised and fabricated for the concurrent degradation of tetracycline and electricity generation. The microchannels within the microfluidic chip, in conjunction with the RhB/PS/Vis all-liquid-phase photocatalytic reaction system, substantially augment the activation of persulfate and the degradation of tetracycline. Moreover, the employment of gold-plated electrodes expedites the conversion of chemical energy derived from the reactions into electricity, accomplishing the conversion of waste into valuable resources.

When the concentration of PS was set at 2 mM and that of sodium sulfate at 1 M, the degradation efficiency within the microfluidic chip was twice that witnessed under normal atmospheric conditions, whereas the electricity generation was threefold greater in comparison to the scenario with a lower sodium sulfate concentration. Finally, the continuous discharge performance of the system was examined, which verified that the microfluidic photocatalytic fuel cell exhibited stable power generation performance. The results suggest that this study proffers a novel methodology for the efficient exploitation of visible light in the degradation of organic pollutants and electricity generation. The microfluidic reactor predicated on the photocatalytic fuel cell erects a new platform for water treatment, proffering an eco-friendly resolution.

## Data Availability

The original contributions presented in the study are included in the article, further inquiries can be directed to the corresponding author.

## References

[1] Huang A., Zhang N., Wang Q.-B., Zhao B.-H., Zhang R.-B., Cheng M., Shi C., Hao X.-D. (2025). Enhanced wastewater treatment using biochar-supported layered-double-hydroxide composites. Chem. Eng. J..

[2] Peng C., Wu Z., Zhang S., Lin B., Nie L., Tian W., Zang H. (2025). Online monitoring of water quality in industrial wastewater treatment process based on near-infrared spectroscopy. Water Res..

[3] Zheng Z., Tian S., Feng Y., Zhao S., Li X., Wang S., He Z. (2023). Recent advances of photocatalytic coupling technologies for wastewater treatment. Chin. J. Catal..

[4] Lu Z., Xu Y., Akbari M.Z., Liang C., Peng L. (2022). Insight into integration of photocatalytic and microbial wastewater treatment technologies for recalcitrant organic pollutants: From sequential to simultaneous reactions. Chemosphere.

[5] Liu Y., Yu Y., Li J., Zhu X., Ye D., Yang Y., Pan Z., Chen R., Liao Q. (2025). Enhanced performance and stability of sulfite/sulfide-assisted photocatalytic fuel cell with CdS@TNTs/Ti mesh photoanode. Chem. Eng. J..

[6] He Y., Chen K., Leung M.K.H., Zhang Y., Li L., Li G., Xuan J., Li J. (2022). Photocatalytic fuel cell—A review. Chem. Eng. J..

[7] He R., Liu L., Shi P., Nie C. (2018). Environmental decontamination using photocatalytic fuel cells and photoelectrocatalysis-microbial fuel cells. J. Chem. Technol. Biotechnol..

[8] Chen Y., Liu Y., Gong X., Wang J. (2025). Recovery of sulfur, generation of electricity and hydrogen peroxide from sulfion-rich wastewater using a novel self-driving photocatalytic fuel cell. Water Res..

[9] Dhawle R., Mantzavinos D., Lianos P. (2021). UV/H_2_O_2_ degradation of diclofenac in a photocatalytic fuel cell. Appl. Catal. B Environ..

[10] Li L., Wang G., Chen R., Zhu X., Wang H., Liao Q., Yu Y. (2014). Optofluidics based micro-photocatalytic fuel cell for efficient wastewater treatment and electricity generation. Lab A Chip.

[11] Zhang H., Wang H., Leung M.K.H., Xu H., Zhang L., Xuan J. (2016). Understanding the performance of optofluidic fuel cells: Experimental and theoretical analyses. Chem. Eng. J..

[12] Wang Y., Chen X., Yao J., Wu J., Gao N., Zhang Z. (2024). Mechanistic insight into the self-catalyzed degradation of Cu-EDTA in photocatalytic fuel cell coupled with peroxymonosulfate. Chem. Eng. J..

[13] Yu T., Yang B., Deng R., Yang T., Jiang J. (2024). The construction of a photocatalytic fuel cell based on piezoelectric-enhanced dual heterojunctions of PVDF–HFP supported 2D/3D composites toward photocatalytic degradation of tetracycline. J. Mater. Chem. A.

[14] Zeng Y., Xu Y., Zhong D., Mou J., Yao H., Zhong N. (2022). Visible-light responsive photocatalytic fuel cell with double Z-scheme heterojunction PTh/Ag_3_PO_4_/BiOI/Ti photoanode for efficient rhodamine B degradation and stable electricity generation. Opt. Mater..

[15] He Y., Yuan R., Leung M.K.H. (2019). Highly efficient AgBr/BiVO_4_ photoanode for photocatalytic fuel cell. Mater. Lett..

[16] Queiroz B.D., Fernandes J.A., Martins C.A., Wender H. (2022). Photocatalytic fuel cells: From batch to microfluidics. J. Environ. Chem. Eng..

[17] Wang C., Liu Y., Han F., Han Y., Liu T., Ren H., Han X. (2023). Nitrogen-doped carbocatalyst activated persulfate (PS) for oxidation polymerization of bisphenol A (BPA): Importance of nonradical activation of PS. Phys. Chem. Chem. Phys..

[18] Wang J., Wang S. (2018). Activation of persulfate (PS) and peroxymonosulfate (PMS) and application for the degradation of emerging contaminants. Chem. Eng. J..

[19] Parvizi T., Parsa J.B., Farnood R. (2022). Synergetic photocatalytic fuel cell and CuFe layered doubled hydroxide as photoactivator of persulfate for dramatically electricity generation of organic pollutants degradation. Appl. Catal. B Environ..

[20] Tang S., Li N., Yuan D., Tang J., Li X., Zhang C., Rao Y. (2019). Comparative study of persulfate oxidants promoted photocatalytic fuel cell performance: Simultaneous dye removal and electricity generation. Chemosphere.

[21] Cai T., Liu Y., Wang L., Dong W., Chen H., Zeng W., Xia X., Zeng G. (2019). Activation of persulfate by photoexcited dye for antibiotic degradation: Radical and nonradical reactions. Chem. Eng. J..

[22] Zhao S., Long Y., Su Y., Wang S., Zhang Z., Zhang X. (2021). Cobalt-Enhanced Mass Transfer and Catalytic Production of Sulfate Radicals in MOF-Derived CeO_2_ • Co_3_O_4_ Nanoflowers for Efficient Degradation of Antibiotics. Small.

[23] Canonica S., Schönenberger U. (2019). Inhibitory Effect of Dissolved Organic Matter on the Transformation of Selected Anilines and Sulfonamide Antibiotics Induced by the Sulfate Radical. Environ. Sci. Technol..

[24] Pirsaheb M., Hossaini H., Janjani H. (2020). Reclamation of hospital secondary treatment effluent by sulfate radicals based–advanced oxidation processes (SR-AOPs) for removal of antibiotics. Microchem. J..

[25] Li J., Li R., Zou L., Liu X. (2019). Efficient Degradation of Norfloxacin and Simultaneous Electricity Generation in a Persulfate-Photocatalytic Fuel Cell System. Catalysts.

[26] Li N., Tang S., Rao Y., Qi J., Zhang Q., Yuan D. (2019). Peroxymonosulfate enhanced antibiotic removal and synchronous electricity generation in a photocatalytic fuel cell. Electrochim. Acta.

[27] Li C., Wan L., Wang N., Chen B., Luo F., Cheng Z., Zhang M. (2022). Photothermal Localization in an Optofluidic Microreactor for Rapid Pretreatment toward Online Pollutant Analysis. ACS Appl. Mater. Interfaces.

[28] Lee W.S., Yeo K.S., Andriyana A., Shee Y.G., Mahamd Adikan F.R. (2016). Effect of cyclic compression and curing agent concentration on the stabilization of mechanical properties of PDMS elastomer. Mater. Des..

[29] Colin C., Levallois P., Botsos-Margerit U., Clément F., Zigah D., Arbault S. (2023). Easy cleaning plus stable activation of glassy carbon electrode surface by oxygen plasma. Bioelectrochemistry.

[30] Pamreddy A., Skácelová D., Haničinec M., Sťahel P., Stupavská M., Černák M., Havel J. (2013). Plasma cleaning and activation of silicon surface in Dielectric Coplanar Surface Barrier Discharge. Surf. Coat. Technol..

[31] Casasanta G., Falcini F., Garra R. (2022). Beer–Lambert law in photochemistry: A new approach. J. Photochem. Photobiol. A Chem..

[32] Rajamani M., Rajan A., Neppolian B. (2023). Photocatalytic pathway toward real time control of tetracycline from industrial wastewater mediated by hetero-structure CuFe_2_O_4_-SnS_2_. J. Environ. Chem. Eng..

[33] Abdulghani A.J., Jasim H.H., Hassan A.S. (2013). Determination of Tetracycline in Pharmaceutical Preparation by Molecular and Atomic Absorption Spectrophotometry and High Performance Liquid Chromatography via Complex Formation with Au(III) and Hg(II) Ions in Solutions. Int. J. Anal. Chem..

[34] Shi Q., Pu S., Yang X., Wang P., Tang B., Lai B. (2022). Enhanced heterogeneous activation of peroxymonosulfate by boosting internal electron transfer in a bimetallic Fe_3_O_4_-MnO_2_ nanocomposite. Chin. Chem. Lett..

[35] Wei L.-Q., Wei J.-B., Yang F., Li Z.-W., Lai H.-F. (2023). Preparation of Thiadiazole Modified UiO-68-CdS Composites for RhB Degradation under Visible Light Irradiation. Crystals.

[36] Gao Y., Zhang Z., Li S., Liu J., Yao L., Li Y., Zhang H. (2016). Insights into the mechanism of heterogeneous activation of persulfate with a clay/iron-based catalyst under visible LED light irradiation. Appl. Catal. B Environ..

[37] Patial S., Sonu, Thakur S., Van Le Q., Ahamad T., Singh P., Nguyen V.-H., Khan A.A.P., Hussain C.M., Raizada P. (2023). Facile synthesis of Co, Fe-bimetallic MIL-88A/microcrystalline cellulose composites for efficient adsorptive and photo-Fenton degradation of RhB dye. J. Taiwan Inst. Chem. Eng..

[38] Du X., Wan J., Jia J., Pan C., Hu X., Fan J., Liu E. (2017). Photocatalystic degradation of RhB over highly visible-light-active Ag_3_PO_4_-Bi_2_MoO_6_ heterojunction using H_2_O_2_ electron capturer. Mater. Des..

